# New insights into the vitamin D requirements during pregnancy

**DOI:** 10.1038/boneres.2017.30

**Published:** 2017-08-29

**Authors:** Bruce W Hollis, Carol L Wagner

**Affiliations:** 1 Department of Pediatrics, Darby Children's Research Institute, Medical University of South Carolina, Charleston, SC, USA

## Abstract

Pregnancy represents a dynamic period with physical and physiological changes in both the mother and her developing fetus. The dramatic 2–3 fold increase in the active hormone 1,25(OH)_2_D concentrations during the early weeks of pregnancy despite minimal increased calcium demands during that time of gestation and which are sustained throughout pregnancy in both the mother and fetus suggests an immunomodulatory role in preventing fetal rejection by the mother. While there have been numerous observational studies that support the premise of vitamin D's role in maintaining maternal and fetal well-being, until recently, there have been few randomized clinical trials with vitamin D supplementation. One has to exhibit caution, however, even with RCTs, whose results can be problematic when analyzed on an intent-to-treat basis and when there is high non-adherence to protocol (as if often the case), thereby diluting the potential good or harm of a given treatment at higher doses. As such, a biomarker of a drug or in this case “vitamin” or pre-prohormone is better served. For these reasons, the effect of vitamin D therapies using the biomarker circulating 25(OH)D is a far better indicator of true “effect.” When pregnancy outcomes are analyzed using the biomarker 25(OH)D instead of treatment dose, there are notable differences in maternal and fetal outcomes across diverse racial/ethnic groups, with improved health in those women who attain a circulating 25(OH)D concentration of at least 100 nmol·L^−1^ (40 ng·mL^−1^). Because an important issue is the timing or initiation of vitamin D treatment/supplementation, and given the potential effect of vitamin D on placental gene expression and its effects on inflammation within the placenta, it appears crucial to start vitamin D treatment before placentation (and trophoblast invasion); however, this question remains unanswered. Additional work is needed to decipher the vitamin D requirements of pregnant women and the optimal timing of supplementation, taking into account a variety of lifestyles, body types, baseline vitamin D status, and maternal and fetal vitamin D receptor (VDR) and vitamin D binding protein (VDBP) genotypes. Determining the role of vitamin D in nonclassical, immune pathways continues to be a challenge that once answered will substantiate recommendations and public health policies.

## Introduction

Pregnancy represents a time of immense change, which includes changes in physical proportions, physiology and responsibility. Arguably, nothing during these times changes more than the requirement and metabolism of vitamin D. Do the current recommendations for the requirements of vitamin D during these critical time periods reflect emerging data? Sadly, no. The minimal recommendations by the Institute of Medicine^1^ of 400–600 IU vitamin D per day and the 0  IU per day recommendation by The World Health Organization,^2^ was echoed by a recent Cochrane Review,^3^ stating that there are simply no requirements for vitamin D during pregnancy, are contrary to expanding published data (later reviewed in this chapter) that suggest otherwise. Why these recommendations continue to be made remains unclear, but clearly not recognizing a problem exists suggests that one does not need to address the “problem”.

During these dramatic times of physiological change, the roles of vitamin D in the pregnant versus, for example, the lactating woman are quite different. In the pregnant women, we believe the primary role of vitamin D to be an immunomodulatory—rather than a calcium-regulating factor, although, it would also retain that function. Further, vitamin D inadequacy in early life is clearly an instance of the “Barker Hypothesis”.^4^ This theory states that certain adult-onset diseases might have their roots in nutritional insults sustained in the perinatal period (either *in utero* or in the early months of infancy or both). Clearly, conditions associated with vitamin D deficiency such as asthma, multiple sclerosis (MS) and other neurological disorders would qualify.^5,6,7,8,9,10,11,12,13,14,15^

If one is reading this review for historical facts presented during the past 50 years of vitamin D recommendations during pregnancy, we suggest you read other recent publications for history in this area.^16,17^ However, if the reader is interested in gaining new insight into the vitamin D requirements and function during pregnancy supported by recent data, we suggest you read on. Also, as a word of caution, with regard to randomized controlled trials (RCT’s) for vitamin D, we discuss the criteria that must be asserted when applied to nutrient research, without which they are largely doomed to fail.^18^ The reasons for this are many and specific cases of this failure will be presented in this text.

## VITAMIN D NOMENCLATURE AND METABOLISM

There are two forms of vitamin D: D_2_ and D_3_. Vitamin D_2_, or ergocalciferol, is made by plants, and fungi such as mushrooms; and vitamin D_3_, or cholecalciferol, is made by animals, including humans, and both are often referred to as the “parent compound”. For the remainder of the review, vitamin D will be used as a reference to both compounds unless otherwise noted.

Vitamin D_3_ is formed in the skin upon exposure to ultraviolet light exposure;^19^ vitamin D_3_ is also acquired through dietary supplementation along with vitamin D_2_ with the amount of this supplementation generally the source of lingering controversy.^1,20,21,22^ Since we are largely a society that avoids sun exposure, the role of dietary supplementation becomes extremely important. Once in the circulation, vitamin D is then converted into 25-hydroxyvitamin D [25(OH)D], which is the major circulating form of the vitamin. This conversion of vitamin D to 25(OH)D is achieved primarily in the liver but can also be achieved in a variety of tissues in an autocrine/paracrine fashion.^23^ Finally, 25(OH)D is converted into the hormonal form of the vitamin—1,25-dihydroxyvitamin D_3_ [1,25(OH)_2_D]—in the kidney for endocrine function and other tissues for autocrine/paracrine function.^23^ This concept is explained in detail elsewhere.^23^

## VITAMIN D METABOLISM DURING PREGNANCY WHEN COMPARED WITH THE NON-PREGNANT STATE

A striking difference exists in vitamin D metabolism during pregnancy and fetal development compared with non-pregnancy and non-fetal states, a point that has been known for at least the past three decades but which has received little attention until recently.^24,25,26,27,28^ The conversion of vitamin D to 25(OH)D appears unchanged during pregnancy, following first-and-zero-order enzyme kinetics.^29^ By contrast, the conversion of 25(OH)D to 1,25(OH)_2_D during pregnancy is unique and unparalleled during life. At no other time during life is 25(OH)D so closely linked with 1,25(OH)_2_D production. By 12 weeks of gestation, 1,25(OH)_2_D serum concentrations are more than twice that of a non-pregnant adult and continue to rise two- to threefold from the non-pregnant baseline rising to over 700 pmol·L^−1^, attaining levels that would be toxic due to hypercalcemia to the non-pregnant individual, but which are essential during pregnancy.^30^ Similarly, in the fetus, cord blood levels of circulating 1,25(OH)_2_D are even more closely tied to fetal levels of 25(OH)D.^31,32^ In neither the mother nor fetus does this conversion seem to be controlled by the classic calcium homeostatic mechanisms during the pregnant state.^30,33^

The rise in circulating 1,25(OH)_2_D levels in the mother/fetus is a remarkable observation. Early-on, the thought was that this increase was to ensure adequate delivery of calcium to the maternal skeleton preservation and fetal skeletal development. Calcium homeostasis, however, is not linked with this increase in 1,25(OH)_2_D, because at 12 weeks of gestation there is no increase in calcium demand by either the mother or fetus. In contrast, this increased concentration of 1,25(OH)_2_D sustained during pregnancy is not sustained during lactation when maternal calcium demand is at least as high as during pregnancy.^34^ Thus, in the mother and fetus during pregnancy, the rise in 1,25(OH)_2_D is dependent on substrate availability—in this case—25(OH)D, and is largely independent of calcium homeostasis.^30^

The control of circulating concentrations of vitamin D, 25(OH)D, and 1,25(OH)_2_D is a complex matter, affected by many disease states such as malabsorption syndromes, aberrant vitamin D metabolism as in sarcoidosis and/or disruptions in the calcium homeostatic system and so on.^35^ Although these are all important, they are beyond the scope of this short review and are not considered further; consideration is given only to what happens to these compounds under normal conditions when vitamin D is obtained through the diet or UV-light induction and how they enter normal cells.

**Figure 1:**
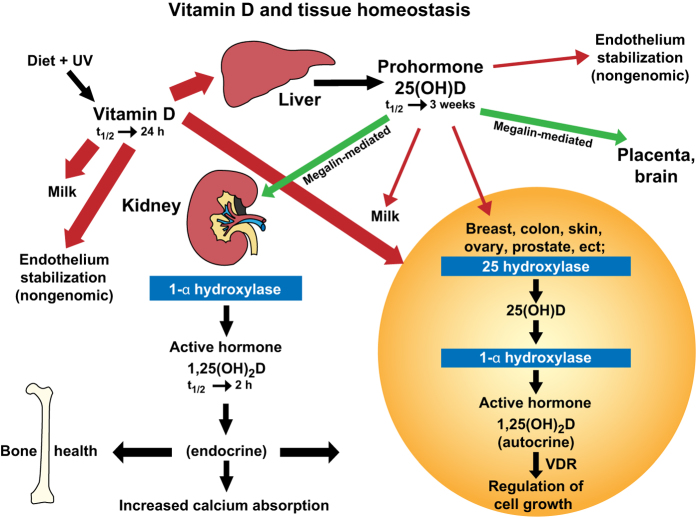
Diagram of the metabolic processes providing vitamin D and its metabolites to various tissues in the body. Tissue distribution of vitamin D and 25(OH)D based on simple diffusion (red arrows) or endocytosis (green arrows). Endocytosis requires the tissue-specific meglin-cubilin system, whereas simple diffusion is primarily controlled by the dissociation constant of the vitamin D compound for the VDBP. Bolder red lines indicate greater diffusion rates due to higher dissociation constant. t_½_, half-life.

In humans, vitamin D_3_ is naturally obtained when sunlight in the UVB range strikes the skin and causes 7-dehydrocholesterol to be converted, following a membrane-enhanced thermal dependent isomerization reaction, into vitamin D_3_, which then diffuses into the circulation through the capillary bed.^36^ Vitamin D also is obtained orally through the diet as either vitamin D_2_ or D_3_. As far as can be determined from the literature, this absorption process is primarily diffusion-based, is dependent on bile acid solubilization, and is not saturable.^37,38,39^ When vitamin D_3_ enters the circulation after UV exposure, it is primarily associated with vitamin D-binding protein (VDBP). In contrast, after intestinal absorption, it is coupled with both VDBP and lipoproteins.^40^ Vitamin D from either route is delivered primarily to the liver, where 25(OH)D is produced, becomes associated with VDBP, and is discharged into the circulation.^41^ Not only circulated to the liver, vitamin D also is circulated to all tissues in the body; many of which are now known to contain both the activating hydroxylase and the vitamin D 25-hydroxylase that converts vitamin D into 25(OH)D, thus achieving autocrine production of 25(OH)D in those tissues^42,43,44,45,46^ (Figure 1). We believe this to be an underappreciated and very important event that has not yet been adequately considered or investigated.

On reaching the circulation, the primary determinant of how long a vitamin D metabolite will stay in circulation is its affinity for VDBP.^47^ Vitamin D, 25(OH)D and 1,25(OH)_2_D have vastly different dissociation constants with regard to VDBP: for 25(OH)D, it is ~10^−9 ^mol, and for vitamin D and 1,25(OH)_2_D, it is ~10^−7^ mol;^48^ in addition, for vitamin D, it is probably reduced to ~10^−8^ mol by its relative insolubility when measured *in vitro*.^49^ These dissociation constants also contribute to the circulating half-lives of these compounds, where for 25(OH)D, it is weeks; for vitamin D, 1 day; and for 1,25(OH)_2_D, a few hours.^50–52^ These dissociation constants also dictate the “free” concentration of compound that is available to enter into cells to be metabolized or to modulate cell activity (Figure 1). In the case of these three compounds, the “free” circulating concentrations are greater for 1,25(OH)_2_D than for intact vitamin D, which in turn is larger than that of 25(OH)D, matching their relative circulating half-lives.

Besides cellular diffusion of free compound, there exists another important tissue—the transport mechanism for these steroids-the megalin-cubilin endocytotic system.^53^ This system is key in the delivery of 25(OH)D to the 25-hydroxyvitamin D-1-α-hydroxylase in the kidney,^54^ which also exists in the parathyroid glands, making its important role in the endocrine function of vitamin D self-evident.^55^ The megalin-cubilin system also functions in the placenta^56^ and brain,^57^ which we will revisit later. Where tissues lack this endocytotic system, however, diffusion of vitamin D compounds in relation to free circulating concentrations becomes inherently important. Interestingly, VDBP-knockout animal models show normal survival when given dietary vitamin D on a daily basis.^58,59^ Because vitamin D metabolite cellular access could only be by diffusion in these animals, this shows that the parent compound vitamin D is normally transferred in wild-type animals through simple membrane diffusion.

Why is calcium metabolism uncoupled from 1,25(OH)_2_D generation during pregnancy and not lactation? One of the leading theories is that 1,25(OH)_2_D is an important immune modulator involved in maternal tolerance to the foreign fetus whose DNA is only half that of the mother’s. Early epidemiological studies involving pregnant women with preeclampsia, a clinical picture of inflammation and vasculitis, vitamin D deficiency has been implicated.^60,61^ Experimental animal models have also strongly suggested vitamin D deficiency as a potential mechanism of placental dysfunction^62,63^ and respiratory maturation.^64^

Vitamin D is a known modulator of inflammation.^65^ Native dietary vitamin D_3_ is thought to be bio-inactive, and the beneficial effects of vitamin D are thought to be largely mediated by 1,25(OH)_2_D.^23^ In many disease states, low circulating 25(OH)D are associated with multiple inflammatory diseases, such as cardiovascular, arthritis, MS, cancer and sepsis.^5,6,7,8,9,10,11,12,14,15,66^ Common to all of these diseases is the disruption of endothelial stability and an enhancement of “vascular leak.” Experimental animal models of preeclampsia clearly demonstrate this endothelial instability leads to placental ischemia.^67^ To that end, Gibson *et al*.^68,69^ have identified vitamin D_3_ as a very effective stabilizer of endothelium and endothelium “leak” through non-genomic mechanisms. This membrane stabilization function is highly structurally specific in that an open b-ring, the cis-triene structure of vitamin D, is required.^68^ This is an incredible new observation. What these studies demonstrate is that vitamin D_3_, 25(OH)D_3_ and 1,25(OH)_2_D_3_ all have the ability to control “endothelial leak”. Most surprising is that on an equal molar basis, vitamin D_3_ is more potent in this function than are 25(OH)D_3_ or 1,25(OH)_2_D.^68^

As shown in Figure 1, this observation is taken one step further. Besides being the most potent stabilizer, vitamin D_3_ would also be the metabolite most accessible to the cell membrane to impart that function. That is because circulating 25(OH)D_3_ is almost totally bound to the VDBP and its “free” concentration so miniscule that there simply is not enough to matter. 1,25(OH)_2_D_3_, while existing in a high circulating “free” form, simply circulates at a level of insignificance for this function. While vitamin D_3_ following its synthesis in the skin would have a slightly longer half-life as opposed to oral dose vitamin D, these differences are minor compared to the half-life of 25(OH)D, which is weeks compared to days.^40^ Given the perceived risks of UV-light exposure, dosing currently in vitamin D studies is by oral supplementation rather than UV-light therapy. Vitamin D_3_, then, if given at physiological doses of 4 000 IU·d^−1^ or greater, would circulate in the “free” form at significant levels and be available to membrane insertion and subsequent endothelial stabilization that is likely to have profound effects on several disease processes. This is truly a new frontier in vitamin D mode of action.

## OBSTETRICAL “PARANOIA” WITH REGARD TO VITAMIN D ADMINISTRATION DURING PREGNANCY

We refer to this type of thinking as “medical lore”; however, in this particular case because it carries forth into current medical care, we view it as dangerous. It happens when medical students are taught something that is based on obsolete data that have carried through to the present. This is absolutely the case with the use of vitamin D during pregnancy. Why is this?

Because of the British experience with idiopathic infantile hypercalcemia attributed to hypervitaminosis D, a terrible, inaccurate association occurred that had a profound effect on the potential of vitamin D supplementation, not only during infancy but also during pregnancy. In 1963, Black and Bonham-Carter^70^ recognized that elfin facies observed in patients with severe idiopathic infantile hypercalcemia resembled the peculiar facies observed in patients with supravalvular aortic stenosis (SAS) syndrome. Shortly thereafter, Garcia *et al*.^71^ documented the occurrence of idiopathic hypercalcemia in an infant with SAS who also had peripheral pulmonary stenosis, mental retardation, elfin facies and an elevated blood concentration of vitamin D. This is an interesting observation because, in 1964, when the article was published, there were no quantitative means of assessing circulating concentrations of vitamin D. In fact, at that time, it was not even proven that vitamin D was further metabolized within the body. By 1967, vitamin D was viewed by the medical community as the cause of SAS syndrome.^72,73^ As a result of the theory that maternal vitamin D supplementation during pregnancy caused SAS syndrome,^74^ animal models were developed to show that toxic excesses of vitamin D during pregnancy would result in SAS.^75,76^ In these earlier cases,^70^ vitamin D had nothing to do with the etiology of SAS. What was described as vitamin D-induced SAS syndrome is now known as Williams Syndrome.^77,78^ Unfortunately, vitamin D intake during pregnancy is still associated with SAS.

Williams Syndrome is a rare developmental disorder and severe genetic affliction related to elastin gene disruption^77^ that is caused by 26–28 elastin and contiguous deleted genes on chromosome 7 g 11.23. This syndrome is characterized by multiorgan involvement (including SAS), dysmorphic facial features, and a distinctive cognitive profile.^78^ Such patients often exhibit abnormal vitamin D metabolism, which makes them susceptible to bouts of idiopathic hypercalcemia.^79^ This relationship was suspected as early as 1976 by Becroft and Chambers.^80^ Subsequently, Taylor *et al.*^81^ demonstrated that children with Williams Syndrome exhibit an exaggerated response of circulating 25(OH)D to orally administered vitamin D. Thus, the fear of vitamin D-induced SAS is based on studies that are no longer valid yet continue to be cited, feared and thus impact treatment.

## OBSERVATIONAL STUDIES SUGGESTING THE FUNCTION OF VITAMIN D EXTENDED BEYOND CALCIUM HOMEOSTASIS DURING PREGNANCY

Again, this review will not discuss the role of vitamin D and skeletal function during pregnancy since this has been discussed endlessly in the past, and truth be told, minimal supplemental vitamin D is required to meet these needs.^1,16,17,82^ Certainly, these needs would be met and exceeded by recommendations we make here with respect to non-skeletal functions of vitamin D.

Beyond skeletal issues, what would these other issues be with respect to vitamin D in pregnancy? To discover what these might be, we rely on associative or observational studies, and in the past 15 years or so many of these studies have been performed. Of high interest is the association of dietary vitamin D_3_ intakes in pregnant women and preeclampsia. Olsen and Secher^83^ point out that in the early 1940s, studies were performed giving pregnant women halibut liver oil, a rich source of vitamin D_3_, with decreases in preterm birth and preeclampsia observed, which the authors attributed to marine n-3 fatty acids, with no mention of vitamin D and its potential effect.^83^ But why would they, since that connection would make no sense to them at the time? As we moved through the recent decades, it became clear that vitamin D and its metabolites’actions in the human body could exist well beyond skeletal events, and thus people started looking at the link between vitamin D and other disease states and conditions.^5,6,7, 8,9,10,11,12,13,14,15,60,61,84,85,86,,87,88,89^Early observational studies uncovered strong relationships between maternal circulating levels of 25(OH)D and preeclampsia,^60,61,84,85^ altered placental vascular pathology,^86^ cesarean section,^87^ glucose tolerance,^88^ adverse birth outcomes due to race,^89^ infection rates,^5^ brain function,^13,14,15^ and respiratory function.^6^ More recent studies have pointed to maternal vitamin D deficiency as a risk factor for abnormal fetal growth patterns, adverse birth outcomes, and reproductive failure.^90,91,92,93,94^ Also, a recent meta-analysis of observational studies has confirmed the fact that maternal vitamin D deficiency increases the risk of preterm birth.^95^

Although public policy cannot be set for supplementation practices based on observational studies, this information is invaluable at pointing research in the direction that could yield public policy changes in vitamin D consumption. These next steps are interventional studies or better yet, randomized clinical trials (RCTs). One has to exhibit caution, however, even with RCTs, whose results can be problematic when analyzed on an intent-to-treat basis and when there is high nonadherence to protocol (as is often the case), thereby diluting the potential good or harm of a given treatment at higher doses. As such, a biomarker of a drug or in this case “vitamin” or preprohormone is better served. For these reasons, analyses of effect of vitamin D therapies using 25(OH)D concentration is a far better indicator of true “effect”.

## RANDOMIZED CONTROLLED TRIALS INVESTIGATING VITAMIN D SUPPLEMENTATION DURING PREGNANCY

Enthusiasm for evidence-based medicine (EBM) has resulted in the extension of its methods to the evaluation of nutrient effects. Heaney^18^ has pointed out EMB, as applied in the evaluation of drugs, is poorly suited to the study of nutrients. In a drug trial, the placebo group will be totally devoid of the compound in question; not so for a nutrient like vitamin D. To perform a true RCT for vitamin D one would have to make sure all subjects were vitamin D-deficient at the study onset. For the duration of the study all subjects would have to remain indoors to avoid any sun exposure. Then and only then could a true RCT be performed for any given function of vitamin D. Of course, what we have suggested here is unethical and is never going to take place. How then does one proceed? Heaney^18^ has provided excellent guidance in this regard. Dr. Heaney proposes five rules for individual clinical studies of nutrient effects. These rules are as follows: (1) basal nutrient status must be measured, used as an inclusion criterion for entry into the study, and recorded in the report of the trial; (2) the intervention must be large enough to change nutrient status and must be quantified by suitable analysis; (3) the change in nutrient status produced in those enrolled in the report of the trial must be measured and reported; (4) the hypothesis to be tested must be that a change in nutrient status produces the sought-for-effect; and (5) co-nutrient status must be optimized in order to ensure that the nutrient is the only nutrition-related, limiting factor in the response. We would add one additional rule to this group, that being: the nutrient in question has to follow an appropriate dosing schedule matching the physiological system being investigated.^23^ Needless to say, while almost all vitamin D RCT’s to this point would fail based on these criteria, evaluation of existing evidence with respect to pregnancy will become the basis for optimizing dietary and clinical recommendations.

Vitamin D supplementation trials involving pregnant women have been performed since 1980.^96^ These early studies were small, did not look at meaningful end points such as accurate bone mineral density measurements (such methods did not exist at that time), or the role of vitamin D on the incidence of such disease states as preeclampsia,^61,84,85,97^ asthma,^98–102^ preterm birth,^95,103,104^ and autoimmune dysfunction,^105–109^ and/or supplemented with nominal doses of vitamin D (0–400 IU·d^−1^).^96^ As a result, no meaningful information or public policy changes occurred because of them. In 2001, our group conceived a large RCT investigating the supplementation of vitamin D to a pregnant population. Our study was radical in design in that we proposed supplementing pregnant women less than 16 weeks of gestation with up to 4 000 IU·d^−1^ vitamin D_3_ until delivery in a double-blind fashion. The goal of the study was to see how much vitamin D was required to raise circulating maternal 25(OH)D concentrations to at least 32 ng·mL^−1^ by the end of gestation. Using mathematical calculations from previous studies, we calculated how much vitamin D_3_ we would need to provide to achieve this endpoint.^110,111^ We selected the 32 ng·mL^−1^ concentration of circulating 25(OH)D based on the suppression of secondary hyperparathyroidism.^112^ We obtained funding from the National Institute of Child Health and Development in 2002; however, because of concerns about the safety of our 4 000 IU·d^−1^ dose of vitamin D_3,_ we had to obtain an investigational new drug application approval from the Food and Drug Administration (FDA; #66 346). This approval was obtained in 2003 and the study began in early 2004. Along with this National Institute of Child Health and Development-sponsored study, we also received funding from the Thrasher Fund to perform a parallel study involving vitamin D supplementation of pregnant women in a community-based format. At the initiation of these RCTs, our end points were safety of the dosing, attained circulating level of maternal 25(OH)D, growth parameters of the infant, and bone-mineral-density of the mother and infant. As for the other factors mentioned in the previous section on vitamin D relationships based on observational studies, those associations had not been made at study initiation, and as a result we had no idea to even look for them, let alone propose them as end points. As such, these end points were analyzed as *post hoc* analyses.

The results of these RCT’s have been presented and published over the past few years.^30,99,104,113–121^ The main finding of these studies was that a 4000 IU·d^−1^ dose of vitamin D_3_ safely elevates circulating 25(OH)D to a level that, regardless of race, fully normalizes vitamin D metabolism and calcium homeostasis in the pregnant women. Using repeated measures, the concentration of 25(OH)D that fully normalized 1,25(OH)_2_D in our study cohort was determined on each subject and plotted to determine the point at which first order kinetics went to zero order.^30^ This point was determined to be when the 25(OH)D concentration was 40 ng·mL^−1^, the production of 1,25(OH)_2_D became substrate independent.^30^ Further, this dose was safe with not a single adverse event observed attributable to vitamin D supplementation.^30,99,104, 113–121^

**Figure 2:**
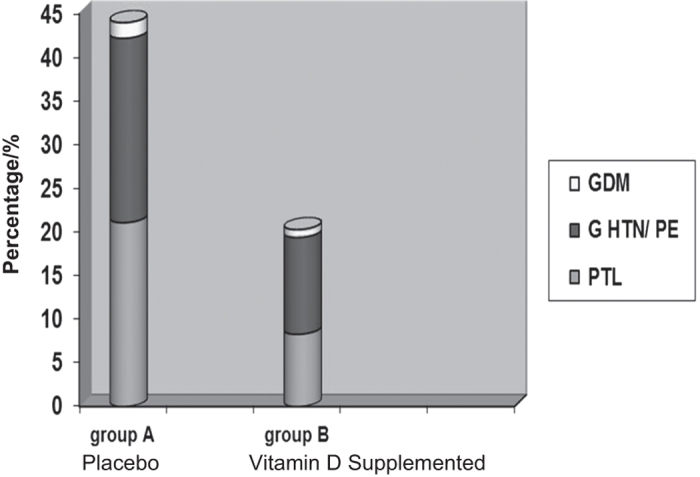
Effect of vitamin D supplementation starting at 20 weeks of pregnancy with respect to the development of complications of pregnancy. Pregnancy complication in the form of preterm labor (PTL), gestational hypertension (GHTN)/preeclampsia (PE) or gestational diabetes mellitus (GDM) were observed in 25/57 (44%) women taking placebo compared to 22/108 (20.4%) women being supplemented with vitamin D. Significance between groups was *P*<0.02. Reproduced with permission from Sablok *et al*.^116^

When our studies were completed in 2009–2010, we were aware of the observational data suggesting favorable efforts of vitamin D on pregnancy outcomes beyond calcium homeostasis. Of course we had collected all of the data on our patient’s outcomes during the trials for safety reasons, and so they were subsequently analyzed. These data were first presented in 2009 at The Vitamin D Workshop in Brugge, Belgium. The data from our studies, when analyzed on an intent-to-treat basis, clearly demonstrated increased vitamin D supplementation decreased complications of pregnancy and C-section births.^30,113^ Further, RCT data and analysis by our group and others have clearly demonstrated that higher doses of vitamin D during pregnancy improve birth outcome data.^104,113^ RCT studies beyond our own have recently demonstrated vitamin D to greatly decrease complications of birth and gestational diabetes,^116,117,120^ aeroallergen sensitization^119^ and markers of regulatory immunity.^121^ The most informative of these RCT studies was performed by Sablok *et al*.^116^ These investigators took a vitamin D-deficient population of pregnant women, with circulating 25(OH)D concentrations of <10 ng·mL^−1^, and supplemented the treatment arm with substantial amounts of vitamin D starting at 20 weeks of gestation. The control group received placebo and thus remained profoundly vitamin D deficient throughout pregnancy. Vitamin D treatment in these women resulted in a substantial decline in the complications of pregnancy (Figure 2). Further, the compliance rate of the women was 100% because the physicians administered the vitamin D to each patient. A study of this type could never be performed in the US as it would be deemed unethical. Be that as it may, it demonstrated the highly significant effect of vitamin D supplementation on the complications of pregnancy. These findings were novel and controversial when they were first presented in 2009 in Belgium.Despite there being high evidence (level 1B) for changing vitamin D dosing and definitions of sufficiency during pregnancy, there was much resistance to change; however, with time and supportive data,these “new” conceptshave prevailed above bias.

## SUPPLEMENTING VITAMIN D DURING PREGNANCY TO PREVENT CHILDHOOD ASTHMA

**Figure 3:**
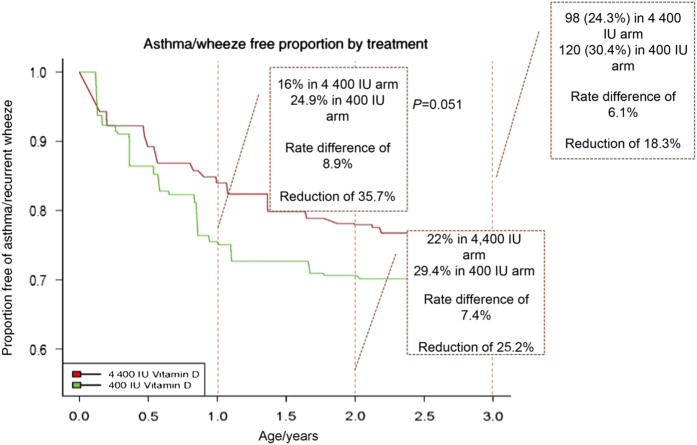
Kaplan–Meier survival estimates for the effect of vitamin D treatment during pregnancy on the development of asthma/recurrent wheeze by age 3 year analyzed in an intent-to-treat format. The hazard ratio for the time to first event of asthma or recurrent wheeze was 0.8 at 3 years, *P*=0.051. Reproduced with permission from Litonjua *et al*.^99^

In 2006, Dr Hollis was contacted by Scott Weiss, MD of the Harvard Medical School with an idea to conduct a RCT using vitamin D supplementation during pregnancy to prevent the development of childhood asthma. Dr Weiss was aware of our ongoing RCT and had excellent observational data suggesting vitamin D supplementation during pregnancy could reduce childhood asthma rates.^122,123^ Subsequently, we obtained funding for this project from The National Institute of Heart, Lung and Blood (NHLBI) and the Vitamin D Antenatal Asthma Reduction Trial (VDAART) was born. In a collaboration between Boston University, Brigham and Women’s Hospital, Harvard Medical School, Kaiser Permanente South California Region, Medical University of South Carolina, Washington University in St. Louis, and NHLBI, a double-blind RCT was performed at three clinical centers: Boston, St. Louis and San Diego; and involved giving supplemental vitamin D_3_ (400 or 4 400 IU·d^−1^) to pregnant women across the three major racial/ethnic groups in the US from 16 weeks of gestation until delivery. The primary endpoint was prevention of asthma/wheeze in the infant/child at 1–3 years post birth. Nearly 900 high-risk subjects were enrolled and completed the study, which recently was published.^99^ The results of this study are quite clear: vitamin D supplementation during pregnancy will decrease asthma or recurrent wheezing rates in children (Figure 3).

A nearly identical RCT study performed in Denmark also recently has been published.^118^ The journal in which these articles appeared—*JAMA*-has attempted to minimize the results and impact of these studies with an Editorial.^124^ In response to this negativity, the authors of these two RCTs performed a meta-analysis (H Wolsk, personal communication). Keep in mind, that a meta-analysis of RCTs is the highest form of validation for therapy/prevention/etiology/harm as defined by The Centre for Evidence-Based Medicine at Oxford University.^125^ The results from these RCTs and meta-analysis studies in women with offspring at high risk for asthma are quite clear: vitamin D_3_ given to a pregnant woman will prevent asthma/wheeze in her child (H Wolsk, personal communication).^99,118^ The mechanisms by which this occurs remain unknown but it is likely that epigenetic *in utero* changes triggered by vitamin D administered to the pregnant women impart functional changes in the fetus.^126–128^

**Figure 4:**
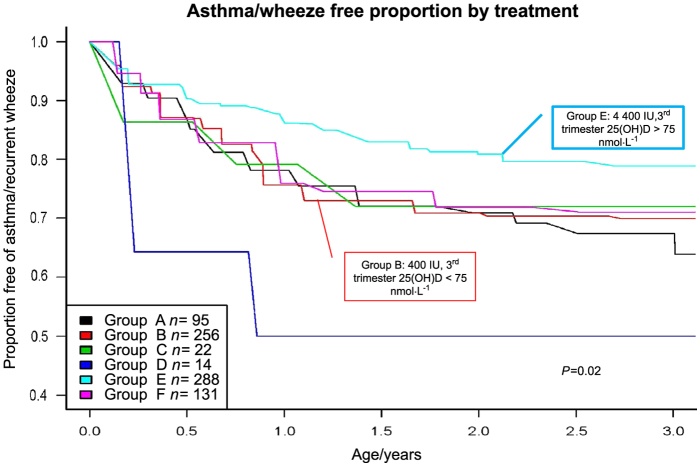
Kaplan–Meier survival estimates for the effect of vitamin D treatment during pregnancy on the development of asthma/recurrent wheeze by age 3 year analyzed stratified by third trimester maternal level of circulating 25(OH)D as an estimate of study compliance. The hazard ratio for the time to first event of asthma or recurrent wheeze now becomes 0.73 at 3 years, *P*<0.02. Reproduced with permission from Litonjua *et al*.^99^

Further analysis of the VDAART study published in *JAMA*^99^ reveal some startling findings. Included in that publication “buried” in the supplemental data is the following: Weiss *et al* have analyzed the data by *post hoc* stratification by maternal third trimester circulating 25(OH)D levels. In this case, circulating 25(OH)D serves as a biomarker of patient compliance for taking supplemental vitamin D during pregnancy. In the *JAMA* publication, adherence or compliance was a huge problem that could not be dealt with in the intent-to-treat study analysis.^99^ This non-adherence was especially acute in the African-American subjects who comprised 43% of the total study subjects and adhered to the prescribed supplementation regimen—as assessed by pill counts and electronic medical cap monitoring—50% of the time. What was the result from this? This non-adherence could bias the study toward null results. While analyses that take into account compliance may have some intrinsic bias as behaviors for taking the study pill could be associated with other behaviors that affect the outcome, nonadherence can significantly affect results of clinical trials, especially in the higher-dose treatment groups where nonadherence can dilute the treatment effect. In these trials, when this bias is factored into the results, the strength of the findings becomes more significant.^98,99^ If one uses circulating 25(OH)D levels as a biomarker of adherence to protocol, the effect of vitamin D preventing childhood asthma becomes highly significant (*P*<0.02; Figure 4).^98,100^ This effect is especially true for the African-American pregnant women in our VDAART study.^101^

## VITAMIN D-INDUCED GENOMIC ALTERATIONS DURING PREGNANCY

**Figure 5:**
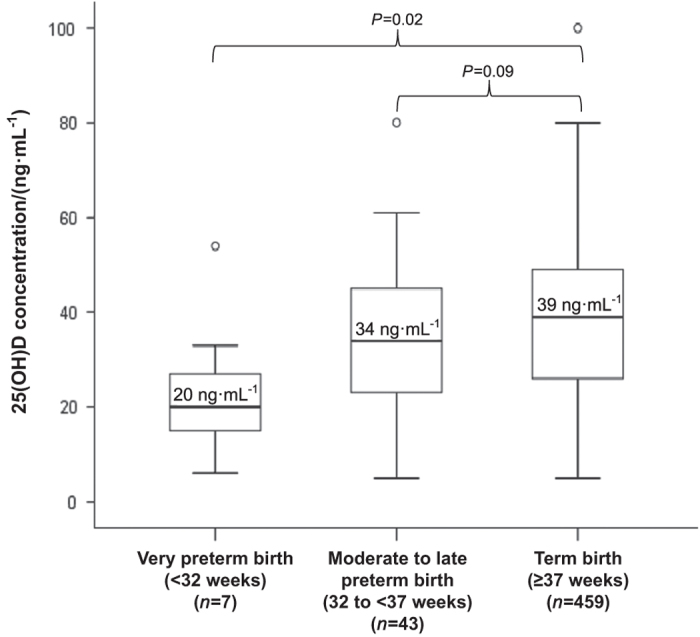
Circulating levels of maternal 25(OH)D with respect to birth staging. Reproduced with permisson from Wagner *et al*.^104^

**Figure 6:**
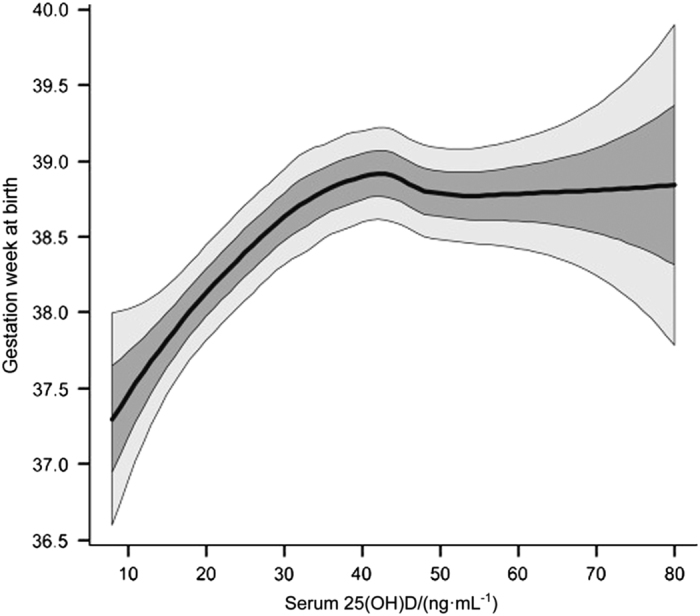
LOESS curve of 25(OH)D concentration and gestational age (weeks) at birth to show the change in average behavior with 1 and 2 s.d. windows superimposed (NICHD and TRF, *n*=509). Black line represents fitted LOESS curve; dark gray area represents 1 s.d.; and light gray area represents 2 s.d. Multivariable log-binomial regression found that 25(OH)D concentrations >40 ng·mL^−1^ reduces the risk of preterm birth by 59% compared to <20.0 ng·mL^−1^, adjusted for covariates. NICHD, National Institute of Child Health and Development. Reproduced with permission from Wagner *et al*.^104^

If one looks at our original pregnancy study from an intent-to-treat fashion, the results are muddled most likely due to nonadherence;^30^ however, taking adherence into account by using circulating 25(OH)D levels as a variable, the true effect on vitamin D supplementation on preterm birth is exposed^104,114^ (Figures 5 and 6). The same associations from our VDAART trial also hold true for the prevention of preeclampsia.^129^ How does this occur? Vitamin D supplementation during pregnancy appears to affect genetic information of several highly functional modules related to systemic inflammation and immune responses and implicates the emergence of a distinctive immune response in women destined to develop preeclampsia.^127,130^

**Figure 7:**
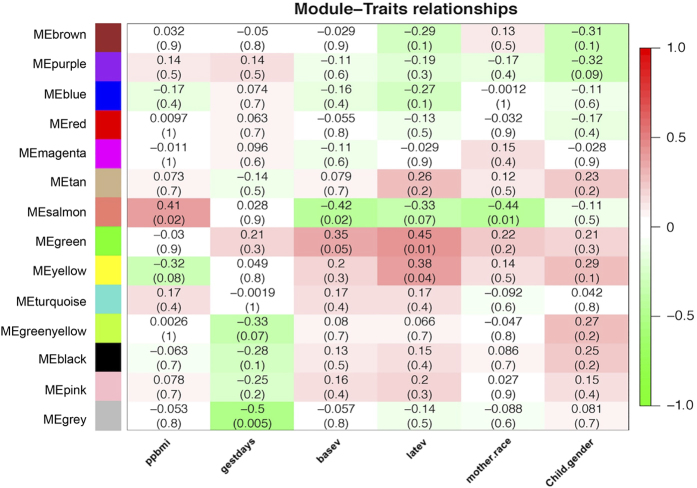
Weighted Co-expression Network Analysis (WGCNA) was carried out on 5839 differentially expressed probes identified by SigGenes. 14 co-expression modules were identified and, correlated to various clinical traits. Gene network, represented by different colored coded co-expression modules (*y* axis) and their association with various clinical traits (*x *axis). The intensity of the colors indicates the strength of the relationships, as indicated by the scale to the right. The range of the scale (+1 to −1) indicates either positive (+1) or negative (−1) correlation with a specific clinical trait. Top number in each box corresponds to the Pearson’s correlation coefficient between a module and a specific trait, while the lower number represents its *P*-value. Traits: ppbmi=pre-pregnancy BMI; gestdays=gestational age; basev=vitamin D levels in first trimester; latev=vitamin D levels in third trimester; mother.race=maternal race (White/African-American); Child.gender+infant gender (boy/girl). Pearson’s correlation (*P*<0.05). Reproduced with permission from Al-Garawi *et al*.^128^

**Figure 8:**
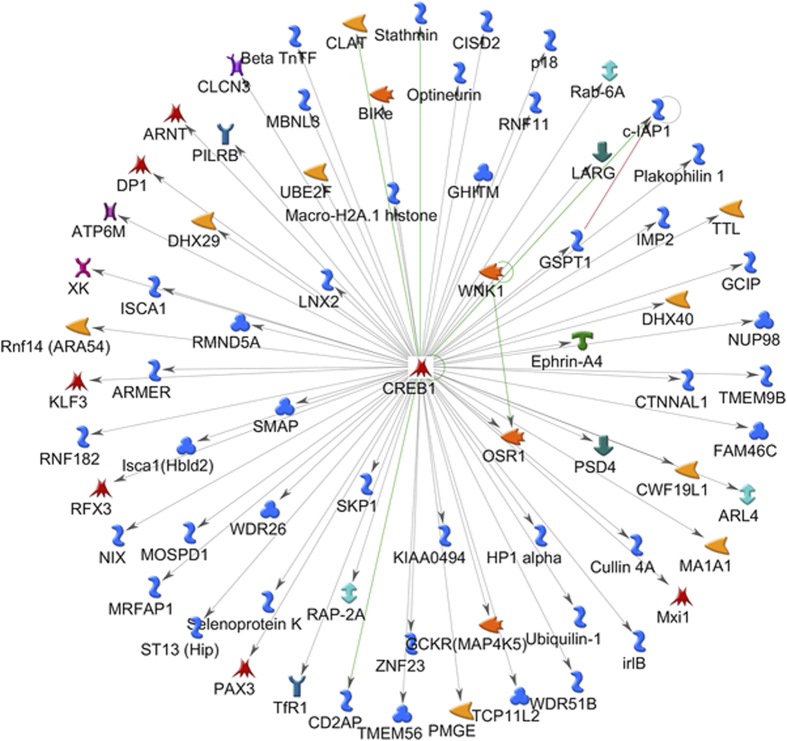
CREB1 transcription factor network depicting CREB1 in center and known interactions among 72 genes demonstrating various functionality within the green module. Hypergeometric test adjusted for multiple comparisons using Benjamini and Hochberg (*P*<0.05). Reproduced with permission from Al-Garawi *et al*.^128^

A recent paper by Al-Garawi *et al*,^128^ derived from the VDAART RCT study, provides direct proof of vitamin D’s ability to induce genomic changes during pregnancy. Patterns of gene expression during human pregnancy are poorly understood. As mentioned earlier, this study was a RCT of vitamin D supplementation in pregnancy for the reduction of pediatric asthma risk.^94^ The trial enrolled 881 women at 10–18 weeks of gestation. Longitudinal gene expression measures were obtained on 30 pregnant women, using RNA isolated from peripheral blood samples obtained in the first and third trimesters. Differentially expressed genes were identified using significance of analysis of microarrays (SAM) and clustered using a weighted gene co-expression network analysis (WGCNA). Gene-set enrichment performed to identify major biological transcriptional profiles between first and third trimesters of pregnancy identified 5 839 significantly differentially expressed genes.Weighted gene co-expression network analysis clustered these transcripts into 14 co-expression modules of which two showed significant correlation with maternal vitamin D levels (Table 1). Pathway analysis of these two modules revealed genes enriched in immune defense pathways and extracellular matrix reorganization as well as genes enriched in notch signaling and transcription factor networks (Table 2 and Figure 7,Figure 8). These data suggest that maternal gene expression changes during pregnancy and that these changes are related to vitamin D supplementation that increase circulating vitamin D levels. What remains unclear is whether these changes in maternal vitamin D levels impact fetal development directly or whether there is any direct effect of maternal gene expression on the fetus.^128^ Another question is whether or not the effects of vitamin D supplementation on gene expression observed in the maternal circulation differ from those effects present in the fetal and maternal placental microenvironment. In addition, the peripheral mononuclear cells themselves are a mixed population with vastly different circulating half-lives that likely have differentially expressed maternal genes. Such factors and issues suggest the need for continued investigation to decipher vitamin D and its metabolites’ dynamic effects on genetic and epigenetic expression during pregnancy that encompasses both the mother and the developing fetus.

Maternal vitamin D supplementation also is involved in epigenetic regulation in children. Pathways affected by DNA methylation, for example, include antigen processing and presentation, inflammation, regulation of cell death, cell proliferation, transmission of nerve impulse, neurogenesis, neuron differentiation, sensory organ development^126^ and vitamin D metabolism.^131^ These modifiable effects of vitamin D on gene expression are truly impressive by any standard.

What is clear from these recent RCTs is that a 4 000 IU·d^−1^ vitamin D_3_ supplement is beneficial to both mother and child and these benefits have nothing to do with the classic role of vitamin D in calcium homeostasis. In fact, maternal hypercalcemia attributed to placental overproduction of 1,25(OH)_2_D has never been reported^30,99^ and the hypercalcemia found in women with molar pregnancies is attributable to PTHrp overproduction.^132,133^ What is not resolved is the dose and time of administration to achieve optimum results. We believe that a target circulating 25(OH)D concentration of 40 ng·mL^−1^ be achieved in pregnancy as early as possible. Because of biochemical heterogeneity in attaining a given concentration of 25(OH)D, we believe all womenshould consume at least 4 000 IU·d^−1^ vitamin D_3_ before conception.^111^

## Neurodevelopment and autoimmune consequences

Can vitamin D deficiency and subsequent vitamin D supplementation during pregnancy impact autoimmune disease and neuropsychological development? There are some compelling data that suggest an association. It has long been thought that the development of MS is a result of a complex interaction between genes and environment with an important environmental factor being vitamin D deficiency.^11^ It is not yet understood how and when vitamin D acts to modulate MS risk, although there is increasing evidence that this occurs through genetic alterations.^10^

Data have emerged that demonstrate that vitamin D supplementation during pregnancy alters transcriptome and epigenetic alterations through DNA methylation in genes that regulate metabolic processes, antigen processing, inflammation, regulation of cell death, cell proliferation, transmission of nerve impulse, neurogenesis, neuron differentiation and sensory organ development.^126^ For now, what we have are observational studies strongly suggesting vitamin D deficiency during pregnancy as a strong causative agent in the development of MS in later life.^7,9^ Agencies such as the Institute of Medicine and Centers of Disease Control will say that this data must be confirmed by RCT. We state here that such an RCT will NEVER be performed because of the cost involved. How do we know that? A few years ago MS world experts were assembled by the National Multiple Sclerosis Society in Chicago to help design such a study. Following two days of meetings, it was determined that the minimum dollar amount to conduct such an RCT would exceed 50 million dollars and would consume their entire budget for at least 5 years. While the study never went forth and never will, other RCTs of treatment to prevent progression of disease for example, may be conducted. Further, an article by Mokry *et al* on Mendelian randomization provides strong support of a causal association of vitamin D and lower MS risk in humans.^134^ Health providers will have to make decisions on the data that are available that come from corollary studies such as these.

An even scarier prospect exists around vitamin D deficiency during pregnancy and neurological disease and altered development.^12–15,135,136,137^ Strong experimental animal evidence points to dire neurological consequences if vitamin D is restricted during pregnancy.^15^ If one wants to read the biochemical basis for this we suggest you read recent reviews by Patrick and Ames.^138,139^ They make an excellent case for intrauterine vitamin D deficiency as it relates to autism, attention deficit disorder, bipolar disorder, schizophrenia and impulse behavior all through the control of serotonin synthesis in the neonatal brain.^138^ There is also a fair amount of observational data available to support these claims.^12–15^ If that is not convincing we suggest you read a recent prospective, interventional vitamin D trial during pregnancy for the prevention of autism in the newborn.^12^ From this data the authors suggest that even performing an RCT would be unethical.^12^

## CURRENT RECOMMENDATION FOR VITAMIN D SUPPLEMENTATION DURING PREGNANCY

At this time, based on RCT data as well as substantial observational and interventional data, we suggest that all pregnant women maintain a circulating 25(OH)D concentration of at least 40 ng·mL^−1^ during the earliest time points of pregnancy.^104^ This will insure maximum protection from pregnancy complications, including preeclampsia in the mother and asthma formation in the infant. To achieve this, intakes of at least 4 000 IU·d^−1^ vitamin D_3_ will be required because of variable individual abilities to convert vitamin D to 25(OH)D.^111^ These supplements have proven to be safe in thousands of patients over the past 15 years, as not a single adverse event due to supplementation has been observed. Further, this level of supplementation lies within the safe intake level as defined by The Endocrine Society.^22^ Finally, does vitamin D qualify as a substance as described by the Barker Hypothesis? The clear answer is yes; it does because its absence during pregnancy imparts detrimental genetic alterations on both mother and fetus.

## Summary

At no other time during the lifespan is vitamin D status more important than during pregnancy, affecting not only the mother but also her growing fetus, and later, her growing infant. While there has been considerable controversy surrounding the daily requirement of vitamin D and what constitutes sufficiency during these critical periods, there is mounting evidence of the importance of vitamin D supplementation during pregnancy to achieve a total circulating 25(OH)D concentration of at least 40 ng·mL^−1^, the point at which the conversion of 25(OH)D to 1,25(OH)_2_D is optimized and associated with a lower risk of comorbidities of pregnancy and better outcomes. Past data suggesting that vitamin D is a teratogenic compound is completely unfounded at the physiological doses reviewed in this chapter. As has been shown, significant amounts of vitamin D—whether their source is sunlight or supplement—are required during pregnancy to protect the mother and fetus and impart genomic imprinting on the fetus to ensure long term health.

With enhanced knowledge about vitamin D’s role as a preprohormone, it is clear that recommendations about supplementation must mirror what is clinically relevant and evidence-based. Future research that focuses on the critical period(s) leading up to conception and during pregnancy to correct deficiency or maintain optimal vitamin D status remains to be studied. In addition, what effects vitamin D has on genetic signatures that minimize the risk to the mother and developing fetus have not been elucidated. Clearly, while there is much more research that needs to be performed, our understanding of vitamin D requirements during pregnancy has advanced significantly during the past few decades.

## Figures and Tables

**Table 1:** Top differentially expressed genes identified by SAM analysis (FDR<0.05)

Entrez ID	Gene	*P*-value	adj. *P*-value	Gene description
*Upregulated genes*				
8451	CUL4A	<0.000 1	<0.000 1	Cullin 4A
83666	PARP9	<0.000 1	<0.000 1	Poly (ADP-ribose) polymerase family, member 9
4128	MAOA	<0.000 1	<0.000 1	Monoamine oxidase A
10935	PRDX3	<0.000 1	<0.000 1	Peroxiredoxin 3
948	CD36	<0.000 1	<0.000 1	CD36 molecule (thrombospondin receptor)
221895	JAZF1	<0.000 1	<0.000 1	JAZF zinc finger 1
6423	SFRP2	<0.000 1	<0.000 1	Secreted frizzled-related protein 2
56994	CHPT1	<0.000 1	<0.000 1	Choline phosphotransferase 1
10935	PRDX3	5.54E−08	7.80E−05	Peroxiredoxin 3
7027	TFDP1	5.54E−08	7.80E−05	Transcription factor Dp-1
4928	NUP98	1.11E−07	9.48E−05	Nucleoporin 98 kDa
440672	NUDT4P1	1.11E−07	9.48E−05	Nudix (nucleoside diphosphate linked moiety X) -type motif4
11171	STRAP	1.11E−07	9.48E−05	Serine/threonine kinase receptor-associated protein
6772	STAT1	1.11E−07	9.48E−05	Signal transducer and activator of transcription 1
140739	UBE2F	1.11E−07	9.48E−05	Ubiquitin-conjugating enzyme E2F

*Downregulated genes*				
5333	PLCD1	<0.000 1	<0.000 1	Phospholipase C, delta 1
6844	VAMP2	<0.000 1	<0.000 1	Vesicle-associated membrane protein 2 (synaptobrevin 2)
6689	SPIB	<0.000 1	<0.000 1	Spi-B transcription factor (Spi-1/PU.1 related)
135	AD0RA2A	<0.000 1	<0.000 1	Adenosine A2a receptor
2788	GNG7	5.54E−08	7.80E−05	Guanine nucleotide binding protein (G protein), gamma 7
3633	INPP5B	5.54E−08	7.80E−05	Inositol polyphosphate-5-phosphatase, 75 kDa
1359	CPA3	5.54E−08	7.80E−05	Carboxypeptidase A3 (mast cell)
84958	SYTL1	1.11E−07	9.48E−05	Synaptotagmin-like 1
26207	PITPNC1	1.11E−07	9.48E−05	Phosphatidylinositol transfer protein, cytoplasmic 1
326624	RAB37	1.11E−07	9.48E−05	RAB37, member RAS oncogene family
128637	TBC1D20	1.11E−07	9.48E−05	TBC1 domain family, member 20
9619	ABCG1	1.11E−07	9.48E−05	ATP-binding cassette, sub-family G
9813	EFCAB14	2.22E−07	0.000 161 771	KIAA0494
9619	ABCG1	2.77E−07	0.000 161 771	ATP-binding cassette, sub-family G
8498	RANBP3	2.77E−07	0.000 161 771	RAN-binding protein 3

Cited from Ref 128, and used with permission from publisher.

**Table 2:** Enrichment analysis of key transcription factors that act on genes in green module (FDR<0.05)

Network	GO processes	Total nodes	Seed nodes	adjusted *P*-value
CREB1	G1/S transition of mitotic cell cycle, metallo-and- iron-sulfur cluster assembly,	73	73	3.980E−193
c-Myc	Modulation by virus of host morphology or physiology or of other organism involved in symbiotic interaction	49	48	8.490E−124
p53	Cell cycle process cellular response to glucose starvation, negative regulation of cell cycle.	20	19	4.240E−48
ZNF143	Single-organism carbohydrate metabolic process, carbohydrate metabolic process, nucleotide metabolic process), nucleoside phosphate metabolic process, CMP-N-acetylneuraminate biosynthetic process.	19	18	1.550E−45
GCR-alpha	Cellular component organization, cellular component organization or biogenesis, cellular amino acid biosynthetic process, rhythmic process, response to arsenic-containing substance.	19	18	1.550E−45
Androgen receptor	Androgen receptor signaling pathway, intracellular steroid hormone receptor signaling pathway, positive regulation of transcription, DNA-dependent-positive regulation of RNA metabolic process, positive regulation of gene expression.	16	15	7.080E−38
SP1	Response to arsenic-containing substance, cellular response to chemical stimulus, cellular nitrogen compound metabolic process, modulation by virus of host morphology or physiology, regulation of transcription from RNA polymerase II promoter in response to hypoxia.	15	14	2.490E−35
ESR1 (nuclear)	Intracellular receptor signaling pathway, RNA metabolic process, intracellular steroid hormone receptor signaling pathway, gene expression, transcription from RNA polymerase II promoter.	15	14	2.490E−35
E2F1	cell cycle process, mitotic cell cycle, negative regulation of cellular process, cell cycle, negative regulation of biological process.	14	13	8.650E−33

Top 10 significantly enriched transcription factor networks of the 202 annotated genes from the green module. For each network, the GO processes are shown along with the number of genes from the green network that are enriched within each network and the number of total nodes that define the network. Total nodes=total number of objects in the network (database); seed nodes=number of objects in green data set. Hypergeometric test adjusted for multiple comparisons using Benjamini & Hochberg (*P*<0.05).

Cited from Ref 128, and used with permission from the publisher.
